# Panelist

**DOI:** 10.1002/alz70856_106091

**Published:** 2026-01-08

**Authors:** Stephen Salloway

**Affiliations:** ^1^ Warren Alpert Medical School, Brown University, Providence, RI, USA

## Abstract

Dr. Salloway will be a panelist.

